# *Baduanjin* exercise for patients with ischemic heart failure on phase-II cardiac rehabilitation (BEAR trial): study protocol for a prospective randomized controlled trial

**DOI:** 10.1186/s13063-018-2759-4

**Published:** 2018-07-16

**Authors:** Meili Yu, Siming Li, Siwei Li, Jingen Li, Hao Xu, Keji Chen

**Affiliations:** 1 Beijing First Hospital of Integrated Chinese and Western Medicine, Beijing, 100021 China; 2 Cardiovascular Diseases Center, Xiyuan Hospital, China Academy of Chinese Medical Sciences, Beijing, 100091 China; 3 Department of Cardiology, Dongzhimen Hospital, The First Affiliated Hospital of Beijing University of Chinese Medicine, Beijing, 100700 China

**Keywords:** *Baduanjin* exercise, Ischemic heart failure, Cardiac rehabilitation, Cardiopulmonary function

## Abstract

**Background:**

Preliminary evidence based on clinical observations suggests that meditative exercise may offer potential benefits for patients with chronic heart failure (CHF). Cardiac rehabilitation (CR), as a class-IA indication in clinical practice guidelines, has been established as an effective strategy to improve quality of life and prognosis of CHF patients. *Baduanjin* exercise is an important component of traditional Chinese *Qigong* exercises. However, its benefits for CHF have not been rigorously tested. We sought to investigate whether *Baduanjin*, as an adjunct to standard care, improves cardiopulmonary function, exercise tolerance, and quality of life in patients with CHF caused by coronary artery disease (CAD).

**Methods/design:**

In this randomized controlled trial, 120 patients will be randomly allocated in a 1:1 ratio to *Baduanjin* exercise combined with conventional exercise of CR (*Baduanjin* exercise group) or conventional exercise of CR alone (conventional exercise group). In addition to conventional physical activity, participants in the *Baduanjin* exercise group will participate in a 45-min *Baduanjin* exercise training session twice a week, for 12 weeks. The primary outcome is walking distance in the 6-min Walk Test (6MWT), and the secondary outcomes are peak oxygen uptake (VO_2_ peak), ventilatory anerobic threshold (VAT), The minute ventilation to carbon dioxide production relationship (VE/VCO_2_ slope), left ventricular end-diastolic volume index (LVEDVi), left ventricular ejection fraction (LVEF), quality of life assessed by the Minnesota Living with Heart Failure Questionnaire (MLHFQ), amino-terminal pro-brain natriuretic peptide (NT-proBNP), hs-CRP, heart rate variability (HRV), New York Heart Association (NYHA) classification, and major adverse cardiovascular events.

**Discussion:**

This is the first trial to evaluate the effects of a *Baduanjin* exercise-based CR program on cardiopulmonary function and exercise tolerance in ischemic CHF patients. If successful, it will prove the value of *Baduanjin* exercise in improving cardiopulmonary function and exercise tolerance in patients with ischemic heart failure on phase-II CR, and may further develop a Chinese *Qigong* exercise-based CR framework.

**Trial registration:**

ClinicalTrials.gov, ID: NCT03229681. Registered retrospectively on 23 July 2017.

**Electronic supplementary material:**

The online version of this article (10.1186/s13063-018-2759-4) contains supplementary material, which is available to authorized users.

## Background

Chronic heart failure (CHF), a terminal stage of various cardiovascular diseases, is characterized by fatigue, chronic reduction of cardiopulmonary function and exercise tolerance with high morbidity and mortality [1–3]. Over 5.7 million Americans have been diagnosed with heart failure, and with the aging population with coronary artery disease (CAD), this number is expected to increase to 8 million by 2030 [2, 3].

Cardiac rehabilitation (CR), a kind of medicine consisting of education, cognitive behavioral therapy, initiative, and positive physical and psychological training, to improve cardiovascular function, is a class-IA recommendation for patients with CHF [4–6]. Although medication, cardiac resynchronization therapy defibrillator (CRT/D) intervention and conventional exercise-based CR are beneficial for CHF and have been advocated as fundamental treatment for CHF, recurrent symptomatic heart failure, high rehospitalization rates and mortality still challenge modern medicine [7]. New approaches are urgently needed to improve quality of life and prognosis in patients with CHF.

*Baduanjin* exercise is a mind-body practice that combines meditation with slow, gentle, and graceful movements, as well as deep breathing and relaxation. It can regulate the vital energy (or *qi*) of collateral channels and organs in the body. As an important component of traditional Chinese *Qigong*, it has been practiced for thousands of years in China as a kind of method of health care. *Baduanjin* exercise involves eight sections of simple and easy-to-learn movements, each benefiting different parts of the body [8]. Therefore, it is a popular and safe community exercise to promote health, which has been recommended by the Chinese Health Qigong Association [9].

Empirical observations have suggested that *Baduanjin* exercise may improve cardiopulmonary function and exercise tolerance [10–12]. A meta-analysis showed that *Baduanjin* exercise can improve left ventricular ejection fraction (LVEF), cardiac output, stroke output and reduce resting myocardial oxygen consumption in elderly patients. Besides, it can also improve the elasticity of vascular, and regulate blood pressure, glucose, and lipid [13–16]. Compared with conventional exercise, *Baduanjin* exercise can additionally relax and ease the mind and spirit, thus improve sleep quality and unhealthy emotions [17]. These features make *Baduanjin* exercise an optimal exercise for patients with CHF, whose daily activity is greatly limited and who suffered a lot physically and mentally after a long period of medical or surgical treatment. Therefore, *Baduanjin* may be a promising exercise for cardiac rehabilitation for CHF.

However, there is a lack of high-quality evidence for the effectiveness of a *Baduanjin* exercise-based cardiac rehabilitation program for patients with ischemic CHF. Therefore, we conduct this randomized controlled trial (RCT) to evaluate the benefits of a *Baduanjin* exercise-based cardiac rehabilitation program in patients with ischemic CHF. We hypothesized that after the 12-week *Baduanjin* practice, patients would have a greater improvement in cardiopulmonary function, exercise tolerance and health-related quality-of-life scores than those practicing conventional exercise alone.

## Methods/design

### Trial oversight

*Baduanjin* exercise for patients with ischemic heart failure in phase-II cardiac rehabilitation (BEAR) trial is a single-center, parallel-design, prospective RCT, and will be conducted at Fuwai Hospital, Chinese Academy of Medical Sciences, China. One hundred and twenty participants are to be recruited and the recruitment is scheduled to begin in May 2017. Participants will be randomly assigned in a 1: 1 ratio to *Baduanjin* exercise combined with conventional exercise (experiment group) or conventional exercise alone (control group) training for 12 weeks. Outcomes will be measured at baseline, 3 months and 6 months by researchers blinded to group allocation. Change in walking distance in the 6-min Walk Test (6MWT) [18, 19] from baseline to 6 months will be served as the primary outcome parameter, whereas the change in exercise capacity index, such as peak oxygen uptake (VO_2_ peak), ventilatory anerobic threshold (VAT), the minute ventilation to carbon dioxide production relationship (VE/VCO_2_ slope) determined by cardiopulmonary exercise test (CPET) [20, 21], LV end-diastolic volume index (LVEDVi), and left ventricular ejection fraction (LVEF) assessed using echocardiography, scores assessed using life quality of the Minnesota Living with Heart Failure Questionnaire (MLHFQ) [22], amino-terminal pro-brain natriuretic peptide (NT-proBNP), high-sensitivity C-reactive protein (hs-CRP), heart rate variability (HRV), major adverse cardiovascular events (MACE), and NYHA classification, will serve as secondary outcome parameters. The study protocol and consent documents have been reviewed and approved by the Ethics Committee of Xiyuan Hospital of China Academy of Chinese Medical Sciences (reference number: 2013XL063–1). If there is any amendment to the protocol, approval must be again sought from the Ethics Committee. The study has been registered at ClinicalTrials.gov (NCT03229681), and the trial will be performed in accordance with the principles of the Declaration of Helsinki and Good Clinical Practice guidelines.

**Fig. 1:**
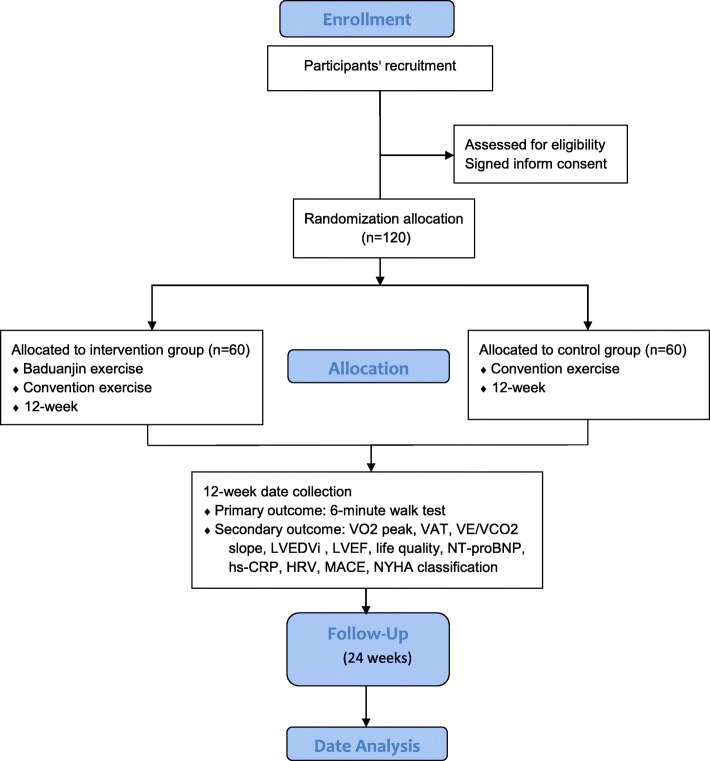
Flow diagram of study design

Study flow of the trial is illustrated in Fig. 1 and described in detail below according to the Consolidated Standards of Reporting Trials (CONSORT) 2010 Statement [23] and the Standard Protocol Items: Recommendations for Interventional Trials (SPIRIT) Checklist (see Additional file 1).

### Diagnostic criteria

The diagnostic criteria of heart failure according to the European Society of Cardiology (ESC) [24] require a combination of typical symptoms and signs, LVEF normal or mildly reduced, left ventricular enlargement but associated structural heart disease (e.g., left ventricular hypertrophy, left atrium enlargement) and/or diastolic heart dysfunction (e.g., E/e’ ≥ 13 or E/A < 1).

Stable patients with CHF were defined as documented heart failure patients with LVEF ≥ 40%, NT-proBNP ≥ 125 pg/ml (or BNP ≥ 35 pg/ml), and with typical clinical symptoms of heart failure, such as breathless, fatigue after activity, which is similar to patients in stage C and NYHA I/II according to the ESC and the American Heart Association (AHA)/ American College of Cardiology Foundation (ACCF) guidelines [25]. The diagnosis of CAD will be based on the standard criteria established in “*Nomenclature and criteria for diagnosis of ischemic heart disease*,” a joint report published by the International Society and Federation of Cardiology and the World Health Organization. In addition, following the current practice of CAD clinical research, we will require subjects to either have a previous history of myocardial infarction or have at least one coronary artery stenosis ≥ 50% confirmed by coronary angiography or by computed tomographic (CT) angiogram to ensure the diagnosis of CAD.

### General inclusion criteria

To participate in this study, subjects must be 40 to 75 years of age, meet the diagnostic criteria of stable CHF and have documented CAD.

In addition, subjects must be able to attend two rehabilitation courses each week at the Cardiac Rehabilitation Center of Fuwai Hospital, China Academy of Medical Sciences. All participants enrolled must provide written informed consent before randomization (for details see Table 1).

**Table 1:** Principal inclusion and exclusion criteria for the BEAR trial

Inclusion criteria	Exclusion criteria
1. Aged from 40 to 75 years	1. Patient’s condition was too severe to exercise, or activity was restrained by other diseases
2. CHF symptoms stable phase (NYHA class I or II) with CHF and with documented CAD	2. Participants with poorly controlled blood pressure (SBP ≥ 180 mmHg or DBP ≥ 100 mmHg)
3. Left ventricular ejection fraction ≥ 40%, NT-proBNP ≥ 125 pg/ml (or BNP ≥ 35 pg/ml)	3. In the acute stage of chronic heart failure
4. Participants signed the informed consent	4. Resting heart rate over 120 beats per minute, or complicated by malignant arrhythmia
	5. Participants having contraindications to cardiopulmonary test or exercise training
	6. Patients complicated by other serious acute or chronic diseases or mental disorders
	7. Participants having practiced any kinds of traditional Chinese medicine exercises in last 3 months

*CHF* chronic heart failure, *DBP* diastolic blood pressure, *NT-proBNP* amino-terminal pro-brain natriuretic peptide, *NYHA* New York Heart Association, *SBP* systolic blood pressure

### General exclusion criteria

Patients having any of the following conditions will be excluded: recent acute heart failure episode (within 1 month), unable to perform rehabilitation exercises for various reasons or having practiced other traditional Chinese *Qigong* exercises (such as *Taijiquan*) in the past 3 months (for details see Table 1).

### Recruitment strategies

Inpatients and outpatients with ischemic CHF in Fuwai Hospital will be screened. Every patient who is interested in, and volunteers to participate in, this trial will be assessed by an attending physician for eligibility. The aim, procedures, and possible side effects of the exercise will be explained in detail to the patients; all patients will be asked to sign a written informed consent form before randomization. No financial incentives will be provided to the attending physicians or patients for enrollment. Eligible patients with initial compliance will proceed to be enrolled.

### Randomization and blinding

We generated a random sequence of 120 numbers using SAS 9.2 software, allocating the patient in a 1:1 ratio to the intervention or the control group. The random sequence will be put into sealed, opaque envelopes by staff not involved with the study to avoid selecting bias. Once a patient meets all the criteria, a random number determining whether the patients will receive *Baduanjin* exercise or not will be delivered to the clinical researchers (the clinical researchers will open an envelope in sequence). Patient allocation will be unblinded only after baseline data is collected for the first phase of the study.

Because *Baduanjin* exercise is an exercise training familiar to the Chinese population, blinding is difficult to achieve at the physician and patient levels. In order to minimize biases as much as possible, all study participants will be discouraged from discussing with one another their practice in the trial. Finally, the clinical researchers will strictly abide by the study’s protocol and will treat the patients in each group with as few differences as possible. Blinding will be maintained at the level of outcome assessment. The individuals performing data management and statistical analyses will not be involved in the clinical procedure of the trials and will not be informed of the treatment allocation.

### Interventions

After recruitment, researchers will evaluate the cardiopulmonary function of participants, educate them about cardiac rehabilitation, and then give them exercise prescription for CR. All the participants will complete 36 CR sessions in CR Center of Fuwai Hospital within 12 weeks.

Participants allocated to the conventional exercise group will receive a closely supervised, group-format aerobic exercise program recommended by current guidelines [6, 26] in the Cardiac Rehabilitation Center for 3 months. The program includes education on diet and nutrition, medication, smoking cessation, physical and mental health, and so on.

In addition to conventional exercise described above, each patient in the intervention group will also receive *Baduanjin* exercise training. Given the low-activity tolerance of CHF patients, we performed with a modified version of *Baduanjin* exercise, in which the difficult movements for CHF patient are removed according to the “Health Qigong *Baduanjin* Standard” enacted by the General Administration of Sports in 2003 and some movements beneficial to cardiopulmonary function for CHF patients are added. They will perform standard training guided by specific *Baduanjin* exercise instructors twice a week, for a total of 12 weeks. Each training session lasts for 45 min, including breathing techniques and mild warm-up (15 min), *Baduanjin* movement (25 min), and relaxation at the end (5 min).

**Fig. 2:**
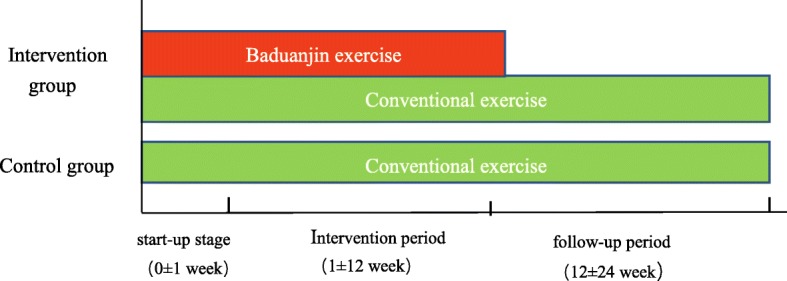
The duration of intervention and followed-up

Researchers will record the subjects’ heart rate and blood pressure before and after training. During the training, exercise intensity will be assessed by the Borg Rating of Perceived Exertion Scale (RPE Scale) [27], which is a frequently used quantitative measure of perceived exertion during exercise. A perceived exertion of 12 or 13, which means “somewhat hard,” is considered the best and at which condition the subjects’ heart rate will be recorded. Subjects will be encouraged to practice B*aduanjin* or conventional exercise at least 30 min a day at home followed the instructional DVD until finishing the follow-up at 6 months. The duration of intervention and followed-up is illustrated in Fig. 2.

### Concomitant treatment

Participants in both groups will continue routine medications, such as anti-platelet and anti-coagulant drugs, angiotensin-converting enzyme (ACE) inhibitors or beta-adrenergic blockers, according to patients’ conditions and maintain their usual treatment visits throughout the study. The doses of ACE inhibitors or angiotensin-receptor blockers and beta-blockers should be gradually increased to target dose whenever possible. All procedures as well as medication prescription will be determined by physicians following the clinical guidelines [28, 29]. The date and reasons of any medical therapy change will be recorded in the case report form (CRF).

### Study outcome measures

The primary outcome is changes in 6-min walking distance assessed by the 6-min Walk Test (6MWT) from baseline to the end of the 12-week intervention. The 6MWT is a quick, safe, and well-tolerated measure that can reflect the functional status in patient with cardiovascular disease.

Secondary outcome includes changes in VO_2_ peak, PVO_2_ and VAT, and VE/ VCO_2_ slope in the cardiopulmonary exercise test (CPET) after the 12-week intervention. CPET is an objective method being increasingly used in a wide spectrum of clinical practice for assessing the functional capacity of CHF patient and has become an important tool to evaluate exercise capacity and predict outcome in patients with heart failure [20].

LVEDVi and LVEF are also to be assessed using echocardiography at baseline and at the end of the 12-week intervention.

MLHFQ, the most widely used scale for assessing the quality of life (QOL) in HF patients, will be used to assess how these two kinds of CR program will affect quality of life of patient with CHF from physical, emotional, social, and mental dimensions.

The serum level of NT-proBNP, an established biomarker that can reflect the cardiac function and stability of heart failure, and hs-CRP, a factor that is independently associated with adverse cardiovascular events, will be also evaluated at baseline and at the end of the 12-week intervention.

MACE, including cardiac death, nonfatal myocardial infarction, documented unstable angina requiring revascularization (bypass surgery or percutaneous coronary intervention), all-cause mortality, re-admission for acute coronary syndrome, heart failure, malignant supraventricular and ventricular arrhythmias influencing hemodynamics, ischemic stroke, and other thromboembolic events during the 6 months’ follow-up will also be recorded.

NYHA classification and the HRV mainly including the SDANN Index and SDNN Index will also be evaluated at baseline and at the end of the 12-week intervention. Table 2 indicates the data to be collected at each phase of the study.

**Table 2:** Study procedures of the BEAR trial

	Phase I: Screening	Phase II: Baseline	Phase III: Month 3	Phase IV: Month 6
Inclusion/exclusion criteria	√			
Signed informed consent	√			
Randomization and allocation	√			
Medical history		√		
Basic characteristic		√	√	√
Physical examination		√	√	√
Medications		√	√	√
Laboratory tests		√	√	
6-min Walk Test		√	√	
Cardiopulmonary exercise test		√	√	
Echocardiography		√	√	
MLHFQ		√	√	√
NYHA classification		√	√	
Incidence of MACE			√	√
Adverse events			√	√
Adherence			√	√
Overall evaluation				√

*MACE* major adverse cardiac events, *MLHFQ* Minnesota Living with Heart Failure Questionnaire, *NYHA* New York Heart Association

### Safety/security assurance

Study participants are monitored weekly during the study intervention for the occurrence of adverse events defined as any undesirable experience. All adverse events during the study will be recorded on an adverse event CRF and will be evaluated for relevance to the intervention. Adverse events will also be reported to the Human Research Committee promptly in accordance with guidelines.

We will strictly follow recommendations from the consensus document of the Heart Failure Association, the European Association for Cardiovascular Prevention and Rehabilitation as well as the exercise standard for testing and training in the consensus from the American Heart Association [30, 31] when conducting Cardiopulmonary exercise testing (CPET). When screening patients, only those eligible and able to finish the test will undergo Cardiopulmonary exercise testing (CPET). Moreover, for enrolled patients meeting the rehabilitation training standard, we stratify them according to the degree of motion risk, and then arrange proper exercise intensity and time according to the risk stratification. Before CR exercise, researchers will educate patients on CHF rehabilitation exercises including training contraindication and notes that should be paid attention to during conventional CR exercise and *Baduanjin* exercise. Moreover, the CR Center of Fuwai Hospital has perfect rescue equipment and we created a thorough contingency plan and rescue procedure for cardiovascular events before the research began. Once adverse events occur during exercise, researchers will immediately start the contingency plan in case of fatal outcomes.

### Data management

The GCP Clinical Center of Xiyuan Hospital will be responsible for randomizing subjects into different groups, monitoring research progress, managing the data, and performing statistical analyses. All investigators involved in data management and analysis will be blinded to treatment allocation. All patient data will be recorded by trained clinical researchers using a standardized, preprinted, and paper CRF. If complete, the CRF will be locked for further revision in preparation for data entry. All CRFs are kept in a secure and lock-protected location. In accordance with our study protocol, extensive procedures are in place to ensure quality control of data entry, missing data minimization, and subject confidentiality. The statistician reviews the database to ensure accurate data collection and correct data export for future analyses. For example, at the GCP Clinical Center, a data manager uninvolved with subsequent data analysis will be responsible for overseeing data entry; to ensure the reliability of the recorded data, two investigators will independently input a copy of the CRF data into a database. A third individual will check the two independently completed database to ensure that they are identical and accurately represented. If the database records are not identical, the data in question will be confirmed from the original CRF, and if any data in the CRF is unclear, the data manager will submit a clarification form to the principal investigator of the study, who will then issue a request for the clinical researchers to resolve it as soon as possible. After blindly checking the database and confirming the accuracy and correctness of the database, we will lock the database for further analysis.

### Statistical analysis

Continuous variables will be presented as the mean ± standard deviation (SD), median or interquartile range (IQR), and categorical variables as frequencies or percentages. Comparability of the baseline data between the two groups will be assessed using a two-sample Student’s *t* test for continuous variables, and the chi-square test or Wilcoxon test for categorical variables. Questionnaire score data, known as non-normally distributed data, will be transformed or analyzed with a nonparametric test.

Analysis of the primary outcome will be conducted according to the intention-to-treat (ITT) principle. The ITT set includes all patients randomized, and the per-protocol set consists of all patients with no major deviation from the protocol and with an adherence rate of 80% or higher at the end of the study. The safety set consists of ITT patients excluding those who receive no CR program or who have no safety record after randomization. We will assess the effect of any missing data on the final results by sensitivity analysis. Dropouts will be included in the analysis by modern imputation methods for missing data.

For all analyses, all tests will be two tailed and a statistical probability of < 0.05 is considered statistically significant. All analyses will be conducted using SPSS 18.0 software (SPSS, Inc., Chicago, IL, USA), unless otherwise noted.

### Adherence

During the 12-week treatment period, participants will be asked to practice strictly according to the CR training prescription, and will not be allowed to take part in any new, additional exercise programs. All patients will be asked to sign a written commitment to guarantee the adherence at baseline evaluation. During the whole study period, subjects will be supervised to record the daily rehabilitation exercise time. Every week, participates will be called to the Cardiac Rehabilitation Center to practice the conventional or *Baduanjin* exercise guided by trained persons. Throughout the 12-week intervention period, the researchers will give patients attendance cards after each session to record the total sessions the patients have taken part in, and patients who missed a class will be asked to attend a “makeup” class. The percentage of compliance will be documented in the CRF. The rate of patient compliance = (total planned number of times − number of absence) / total number of times × 100%. A compliance rate of greater than 80% is considered as good. Otherwise, less than 80% is considered as poor. Patients will be asked to visit the CR Center regularly according to the study protocol, and researchers will inform them of the visit in advance. Attendance of less than 20% is considered as dropout.

### Sample size calculation

Sample size is calculated, based on the 6MWT, according to the following superiority test formula:

$$ {n}_c=\frac{\left(1+\frac{1}{k}\right){\left({\mu}_{1-\alpha }+{\mu}_{1-\beta}\right)}^2{\sigma}^2}{{\left[\left({x}_t-{x}_c\right)-\varDelta \right]}^2}, $$

*n*_*t*_ = *kn*_*c*_, The patients’ numbers were arranged in equal proportion (*k* = 1), a superiority one-sided test was employed. According to result of a previous study [32], *x*_t_ = 512.5, *x*_c_ = 436.2, *δ*_t_ = 50.2, *δ*_c_ = 49.8, *δ* is the estimated standard difference calculated as 50.0, and *Δ* = 0.9, *δ* = 45.0. Setting a type I error rate of alpha at 0.05, *μ*_1-a_= 1.64, and setting a type II error rate of beta at 0.1 (a power of 90%), *μ*_1-β_ = 1.282, the calculated sample size of each group is around 49. Assuming conservatively that 20% may be lost to follow-up within 6 months, the total sample size needed to detect this difference at a 5% level of significance with a power of 90% is 118 patients. For our study, we decided to recruit 120 patients for the convenience of randomization.

## Discussion

We conducted this trial by trying to assess whether the addition of *Baduanjin* to conventional CR exercise will further improve exercise endurance, reserve of heart function and quality of life, and, if successful, this trial should provide a promising exercise program for CR. As a matter of fact, the building and development of CR is still under exploration, and currently, CR, mainly consists of contemporarily popular exercise. As a traditional Chinese *Qigong*, *Baduanjin* has long been practiced for health care since it has the function of regulating organs and dredging the channels and collaterals [10, 11]. This exercise is especially suitable for patient with CHF complicated with CAD whose activity tolerance is poor because the action of *Baduanjin* exercise is simple and gentle. However, its effect has not been confirmed by objective evaluation.

There are several strengths of our trial. First of all, we use the objective and quantitative Cardiopulmonary exercise testing (CPET) to assess the improvement of cardio-pulmonary function, which is reliable and convincing. Second, *Baduanjin* exercise is easy to master and gentle to practice for CHF patients, which encourages compliance. Moreover, if proved to be effective, the costless *Baduanjin* could be a widely welcomed exercise for CR. On the one hand, it saves time and facilitates for CHF patients who have poor activity tolerance. On the other hand, it may have the effect of improving the prognosis for patients with ischemic CHF, finally it may reduce the social medical expenditure.

A potential limitation of the BEAR trial is that participants and exercise coaches cannot be blinded because it is difficult to conduct blinding in non-pharmacological trials [33]. It is inevitable that performance bias always exists in a practical trial. However, the exercise coaches will not participate in the procedure of recruitment, assessment and data analysis of this study which will minimize bias.

In summary, the BEAR trial will be the first RCT to evaluate the impact of *Baduanjin* exercise on activity tolerance in patients with ischemic CHF and the results of this trial will help to establish the optimal approach for treating patients with CHF and provide reliable evidence for the application of *Baduanjin* exercise in cardiac rehabilitation.

## Trial status

This trial begun to recruit patients from May 2017, and there have been 768 volunteers screened up to October 2017, of which 68 participants were included and 43 of them have completed the 12-week intervention. No serious adverse events (SAEs) have occurred to date.

## Additional file

Additional file 1: Standard Protocol Items: Recommendations for Interventional Trials (SPIRIT) Checklist. (PDF 131 kb)

## References

[1] Mozaffarian D, Benjamin EJ, Go AS (2016). Heart disease and stroke Statistics—2016 update: a report from the American Heart Association. Circulation.

[2] Ziaeian B, Fonarow GC (2016). Epidemiology and aetiology of heart failure. Nat Rev Cardiol.

[3] Heidenreich PA, Albert NM, Allen LA (2013). Forecasting the impact of heart failure in the United States: a policy statement from the American Heart Association. Circ Heart Fail.

[4] Piepoli MF, Hoes AW, Agewall S (2016). 2016 European guidelines on cardiovascular disease prevention in clinical practice: the Sixth Joint Task Force of the European Society of Cardiology and Other Societies on cardiovascular disease prevention in clinical practice (constituted by representatives of 10 societies and by invited experts): developed with the special contribution of the European Association for Cardiovascular Prevention and Rehabilitation (EACPR). Eur Heart J.

[5] Yancy CW, Jessup M, Bozkurt B (2013). 2013 ACCF/AHA guideline for the management of heart failure: a report of the American College of Cardiology Foundation/American Heart Association Task Force on Practice Guidelines. J Am Coll Cardiol.

[6] American Association of Cardiovascular & Pulmonary Rehabilitation. Guidelines for Cardiac Rehabilitation and Secondary Prevention Programs, Fifth Edition. United States: Human Kinetics; 2013.

[7] Townsend N, Nichols M, Scarborough P (2015). Cardiovascular disease in Europe: epidemiological update 2015. Eur Heart J.

[8] Koh TC (1982). *Baduanjin*: an ancient Chinese exercise. Am J Chin Med.

[9] Gong BM. The value analysis of *Baduanjin* in international cultural vision. Chin Med Cult. 2016;4:37–40.

[10] Qin G (2012). Effect of *qigong* on cardiovascular function in college students. J Wuhan Inst Phys Educ.

[11] Zeng YG, Zhou XQ, Wang AL (2005). Research on the impacts of fitness *qigong Baduanjin* on figure and physical function among the middle-aged and aged people. J Beijing Sport Univ.

[12] Wang Y (2011). The influence of health *qigong Baduanjin* training on the psychological health of college students. J Beijing Sport Univ.

[13] Wang XQ, Pi YL, Chen PJ (2016). Traditional Chinese exercise for cardiovascular diseases: systematic review and meta-analysis of randomized controlled trials. J Am Heart Assoc.

[14] Xiong X, Wang P, Li S (2015). Effect of *Baduanjin* exercise for hypertension: a systematic review and meta-analysis of randomized controlled trials. Maturitas.

[15] Luskin FM, Newell KA, Griffith M (1998). A review of mind-body therapies in the treatment of cardiovascular disease, part 1: implications for the elderly. Altern Ther Health Med.

[16] Chen BL, Guo JB, Liu MS (2015). Effect of traditional Chinese exercise on gait and balance for stroke: a systematic review and meta-analysis. PLoS One.

[17] Chan JSM, Ho RTH, Chung K-F (2014). *Qigong* exercise alleviates fatigue, anxiety, and depressive symptoms, improves sleep quality, and shortens sleep latency in persons with chronic fatigue syndrome-like illness. Evid Based Complement Alternat Med.

[18] Guyatt GH, Sullivan MJ, Thompson PJ (1985). The 6-minute walk: a new measure of exercise capacity in patients with chronic heart failure. Can Med Assoc J.

[19] Zugck C, Krüger C, Dürr S (2000). Is the 6-minute walk test a reliable substitute for peak oxygen uptake in patients with dilated cardiomyopathy?. Eur Heart J.

[20] Kao W, Jessup M (1994). Exercise testing and exercise training in patients with congestive heart failure. J Heart Lung Transplant.

[21] Gerardi DA, Lovett L, Benoit-Connors ML (1996). Variables related to increased mortality following out-patient pulmonary rehabilitation. Eur Respir J.

[22] Rector TS, Tschumperlin LK, Kubo SH (1995). Use of the Living With Heart Failure Questionnaire to ascertain patients’ perspectives on improvement in quality of life versus risk of drug-induced death. J Card Fail.

[23] Moher D, Hopewell S, Schulz KF (2010). CONSORT 2010 explanation and elaboration: updated guidelines for reporting parallel group randomised trials. BMJ.

[24] Catapano AL, Graham I, De Backer G (2016). 2016 ESC/EAS Guidelines for the Management of Dyslipidaemia: The Task Force for the Management of Dyslipidaemia of the European Society of Cardiology (ESC) and European Atherosclerosis Society (EAS) developed with the special contribution of the European Association for Cardiovascular Prevention and Rehabilitation (EACPR). Eur Heart J.

[25] Yancy CW, Jessup M, Bozkurt B (2013). 2013 ACCF/AHA guideline for the management of heart failure: a report of the American College of Cardiology Foundation/American Heart Association Task Force on practice guidelines. Circulation.

[26] Haskell WL, Lee IM, Pate RR (2007). Physical activity and public health: updated recommendation for adults from the American College of Sports Medicine and the American Heart Association. Circulation.

[27] Borg GA. Psychophysical bases of perceived exertion. Med Sci Sports Exerc. 1982;14(5):377–81. 7154893

[28] Fletcher GF, Ades PA, Kligfield P (2013). Exercise standards for testing and training: a scientific statement from the American Heart Association. Circulation.

[29] Piepoli MF, Conraads V, Corrà U (2011). Exercise training in heart failure: from theory to practice. A consensus document of the Heart Failure Association and the European Association for Cardiovascular Prevention and Rehabilitation. Eur J Heart Fail.

[30] Piña IL, Apstein CS, Balady GJ (2003). Exercise and heart failure: a statement from the American Heart Association Committee on exercise, rehabilitation, and prevention. Circulation.

[31] American College of Sports Medicine (2000). ACSM’S guidelines for exercise testing and prescription[M].

[32] Xiong XH, Deng X (2016). The clinical observation of *Baduanjin* for chronic heart failure with CHD. Mod Med J China.

[33] Boutron I, Moher D, Altman DG, et al CONSORT Group. Extending the CONSORT statement to randomized trials of nonpharmacologic treatment: explanation and elaboration. Ann Intern Med 2008; 148: 295–309. 10.7326/0003-4819-148-4-200802190-00008 18283207

